# Indocyanine Green as a Photosensitizer in Periodontitis Treatment: A Systematic Review of Randomized Controlled Trials

**DOI:** 10.3390/life15071015

**Published:** 2025-06-25

**Authors:** Rafał Wiench, Jakub Fiegler-Rudol, Katarzyna Latusek, Katarzyna Brus-Sawczuk, Hanna Fiegler, Jacek Kasperski, Dariusz Skaba

**Affiliations:** 1 Department of Periodontal Diseases and Oral Mucosa Diseases, Faculty of Medical Sciences in Zabrze, Medical University of Silesia, 40-055 Katowice, Poland; katarzyna.latusek@sum.edu.pl (K.L.); dskaba@sum.edu.pl (D.S.); 2 Comprehensive Dentistry Department, Medical University of Warsaw, Binieckiego 6, 02-097 Warszawa, Poland; katarzyna.brus-sawczuk@wum.edu.pl; 3 Department of Dental Prosthetics, Faculty of Medical Sciences in Zabrze, Medical University of Silesia, 40-055 Katowice, Poland; hfiegler@sum.edu.pl (H.F.); jkasperski@sum.edu.pl (J.K.)

**Keywords:** indocyanine green, photodynamic therapy, periodontitis, antimicrobial therapy, diode laser, adjunctive periodontal treatment, clinical attachment level, probing depth

## Abstract

Photodynamic therapy (PDT) using indocyanine green (ICG) has gained attention as an adjunctive treatment for periodontitis due to its antimicrobial and anti-inflammatory properties and its ability to penetrate deep periodontal tissues via near-infrared light activation. We aimed to evaluate the clinical and microbiological efficacy of ICG-mediated PDT as an adjunct to conventional periodontal therapy in patients with periodontitis based on data from randomized controlled trials (RCTs). A systematic search of PubMed, Embase, Scopus, and the Cochrane Library was conducted to identify randomized controlled trials (RCTs) exclusively investigating ICG-PDT in periodontitis based on predefined eligibility criteria. Studies were selected based on predefined inclusion criteria, and methodological quality was assessed using a 14-point scoring system. Data were extracted on clinical outcomes (e.g., probing depth, clinical attachment level) and microbiological changes. Sixteen RCTs met the inclusion criteria. Most studies reported improvements in probing depth, clinical attachment level, and microbial reduction following ICG-aPDT; however, some trials found no significant differences compared to control groups. These discrepancies may be attributable to variations in laser settings, ICG concentration, treatment frequency, or initial disease severity. Microbiological benefits included significant reductions in key periodontal pathogens. The therapy was well tolerated, with no adverse effects reported. However, variability in treatment protocols and limited long-term follow-up restricted the ability to draw definitive conclusions. ICG-mediated PDT is a promising, safe, and effective adjunct in periodontal therapy. Future trials should aim for protocol standardization and long-term outcome assessment to strengthen clinical guidance.

## 1. Introduction

Periodontitis is a prevalent chronic inflammatory disease that affects the supporting structures of the teeth, including the periodontal ligament, cementum, and alveolar bone [1,2,3]. If left untreated, periodontitis leads to progressive tissue destruction, tooth mobility, and ultimately, tooth loss [3]. Beyond its impact on oral health, periodontitis has been increasingly linked to a range of systemic conditions, including cardiovascular diseases, diabetes mellitus, respiratory diseases, and adverse pregnancy outcomes, highlighting the importance of effective management strategies [4,5,6]. The primary etiological factor in periodontitis is the presence of a complex polymicrobial biofilm on the tooth surface and within periodontal pockets [7]. Conventional periodontal therapy, particularly scaling and root planing (SRP), remains the gold standard for mechanical disruption and removal of subgingival biofilms and calculus deposits [8,9]. However, mechanical instrumentation alone may not always achieve complete eradication of pathogenic bacteria, particularly in deep or anatomically complex periodontal pockets, furcation areas, and root concavities [10]. Residual bacterial load can contribute to persistent inflammation, disease progression, and compromised treatment outcomes [11]. In response to these limitations, adjunctive antimicrobial therapies have been explored to enhance periodontal treatment efficacy [12]. Systemic and local antibiotics have demonstrated adjunctive benefits; however, concerns regarding antibiotic resistance, allergic reactions, and disruption of the commensal microbiota have driven interest in alternative non-antibiotic antimicrobial strategies [13]. Photodynamic therapy (PDT) has emerged as a promising adjunctive modality in this context, offering targeted antimicrobial effects with minimal systemic impact and a low risk of developing resistance [14]. PDT involves the application of a photosensitizing agent, which preferentially accumulates in bacterial cells [15]. Upon activation by light of a specific wavelength, the photosensitizer undergoes a photochemical reaction, generating reactive oxygen species (ROS), such as singlet oxygen and free radicals [16]. ROS exert potent cytotoxic effects, leading to bacterial cell wall disruption, membrane damage, and ultimately bacterial cell death [17]. Importantly, PDT can also modulate the host immune response and reduce local inflammation, potentially contributing to enhanced periodontal healing. Among the various photosensitizers investigated for periodontal applications, indocyanine green (ICG) has garnered increasing attention [18,19]. ICG is a water-soluble tricarbocyanine dye approved by the U.S. Food and Drug Administration (FDA) for clinical use in medical imaging [20,21]. It exhibits favorable properties for photodynamic applications, including high photostability, low toxicity, and strong absorption of near-infrared (NIR) light around 800 nm [22]. The use of NIR light allows for deeper tissue penetration compared to visible light wavelengths, which may enable more effective targeting of subgingival pathogens located within periodontal pockets and tissues [23]. Recent studies have explored the potential of ICG-mediated PDT as an adjunct to SRP in the treatment of periodontitis [24]. Given the growing interest in PDT and the need for evidence-based guidance on its clinical application, a comprehensive synthesis of the current literature is warranted. This systematic review aims to critically evaluate and summarize the available evidence from randomized controlled trials investigating the use of indocyanine green as a photosensitizer in periodontal therapy. Specifically, it seeks to assess the effectiveness of ICG-mediated PDT in reducing bacterial load and improving periodontal outcomes compared to conventional treatments or other photosensitizers. By systematically analyzing the methodological quality, treatment protocols, and clinical outcomes reported in the included studies, this review aims to clarify the role of ICG-PDT in the contemporary management of periodontitis and identify directions for future research.

## 2. Materials and Methods

### 2.1. Focused Question

The systematic review was designed based on the PICO framework [25], structured to address the following question: In patients with periodontitis (Population), does treatment involving indocyanine green (ICG)-mediated photodynamic therapy (Intervention), compared to conventional periodontal therapies, alternative photosensitizers, or standard care without photodynamic treatment (Comparison), lead to greater improvements in bacterial reduction and periodontal outcomes (Outcome)?

### 2.2. Search Strategy

**Figure 1:**
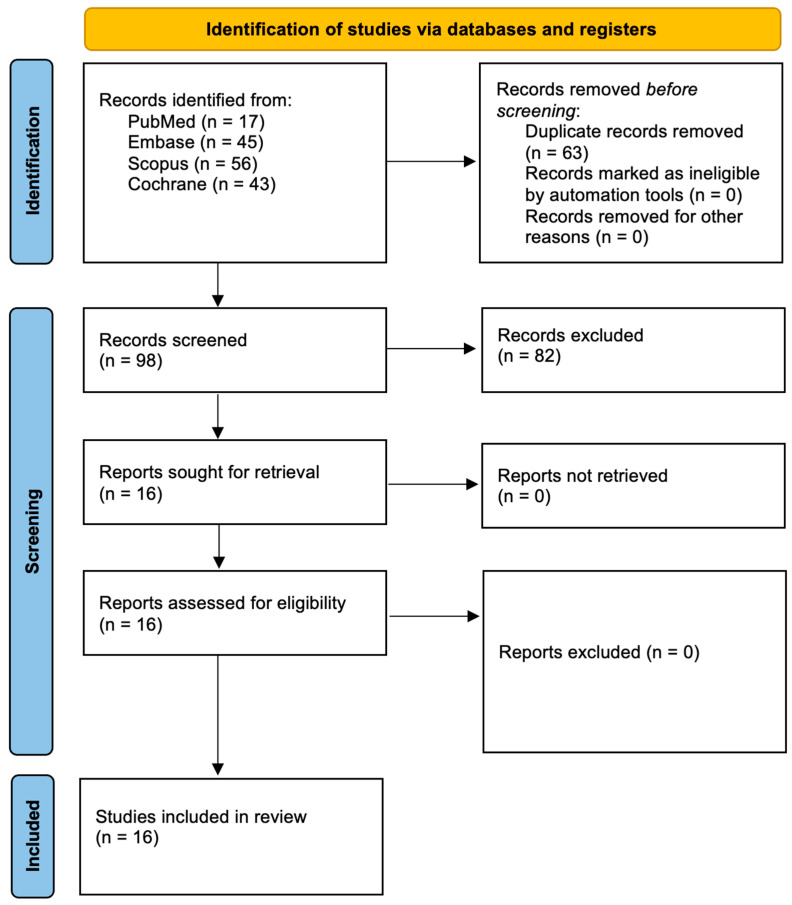
PRISMA 2020 flow diagram.

This systematic review, registered in PROSPERO (ID: CRD420251040973), was conducted in accordance with the PRISMA 2020 guidelines to ensure transparent and systematic reporting [26]. A comprehensive literature search was performed across major databases, PubMed/Medline, Embase, Scopus, and the Cochrane Library, to identify randomized controlled trials (RCTs) evaluating the use of indocyanine green as a photosensitizer in the treatment of periodontitis (Table 1). The complete search strategy is illustrated in Figure 1. Three independent reviewers executed the database queries using a predefined set of search terms focused on periodontitis management and photodynamic therapy with ICG. Studies were limited to those published in English, with no restrictions on publication date. The study selection process involved initial screening of titles and abstracts based on eligibility criteria (outlined in Table 2), followed by independent full-text reviews conducted by two reviewers. A snowballing method was also employed, screening the reference lists of included studies to identify any additional relevant trials. The final search was conducted on 15 March 2025 and included studies published from 1 January 2015 to 15 March 2025.

### 2.3. Study Selection Process

To uphold methodological rigor and reduce potential bias, all retrieved records were subjected to a structured, independent screening process by multiple reviewers. Titles and abstracts were carefully assessed against predefined inclusion criteria. Any discrepancies in study selection were resolved through discussion to ensure consistency in decision-making. The eligibility criteria were specifically tailored to capture high-quality randomized controlled trials evaluating the antimicrobial effectiveness of indocyanine green-mediated photodynamic therapy in the treatment of periodontitis.

### 2.4. Risk of Bias in Individual Studies

To ensure objectivity and minimize the risk of selection bias, the initial screening of titles and abstracts identified through the search strategy was performed independently by multiple reviewers. Inter-rater agreement was assessed using Cohen’s kappa coefficient, providing a quantitative measure of consistency between reviewers [27]. In cases where discrepancies arose regarding study eligibility, they were resolved through structured discussion until full consensus was reached. This systematic and transparent approach was implemented to uphold the methodological rigor of the review and guarantee the accurate identification of relevant randomized controlled trials.

### 2.5. Quality Assessment

The methodological quality of the included studies was independently assessed by three reviewers, focusing on key elements related to the design, execution, and reporting of indocyanine green (ICG)-mediated photodynamic therapy (PDT) interventions. A structured scoring system was employed to evaluate risk of bias, assigning 1 point for each criterion met (“yes”) and 0 points for unmet items (“no”), across fourteen predefined domains: (1) clear reporting of ICG preparation and concentration (e.g., dye concentration, solvent, stability); (2) identification of the light source used for activation (e.g., diode laser, LED, specific manufacturer); (3) comprehensive description of the irradiation protocol, including light parameters (wavelength, power output, energy density), exposure time, and treatment area; (4) provision of full technical specifications of the light delivery system (spot size, continuous or pulsed mode, energy fluence); (5) confirmation of photosensitizer–light interaction (e.g., timing between ICG application and light exposure, verification of dye uptake); (6) inclusion of an appropriate control group (e.g., scaling and root planing only, placebo, light, or ICG alone); (7) use of valid statistical analysis for clinical (e.g., probing depth, clinical attachment level) and/or microbiological outcomes; (8) transparent outcome reporting without selective reporting or missing outcome data; (9) disclosure of conflicts of interest and funding sources; (10) randomization process (e.g., method of random sequence generation, allocation concealment); (11) lack of deviations from intended interventions (e.g., compliance with assigned interventions, blinding of participants and personnel); (12) no missing outcome data (e.g., incomplete follow-up, reasons for missing data); (13) no bias in measurement of the outcome (e.g., blinding of outcome assessors, objective outcome measurements); and (14) no bias in selection of the reported result (e.g., selective reporting of outcomes or analyses).

The scoring system was adapted from the Cochrane Risk of Bias Tool (RoB 2.0) and supplemented with technical criteria relevant to photodynamic therapy (e.g., light parameters, ICG concentration, and photosensitizer–light interaction), consistent with previous systematic reviews in dental laser therapy [28]. Each study was assigned a total score out of fourteen, and the risk of bias was categorized as high (0–5 points), moderate (6–10 points), or low (11–14 points). Discrepancies between reviewers were resolved by consensus or, if necessary, by consultation with a fourth reviewer. This evaluation process was conducted in accordance with the recommendations of the Cochrane Handbook for Systematic Reviews of Interventions [28]. Table 3 shows the results of the quality assessment.

### 2.6. Data Extraction

After reaching consensus on the final set of studies for inclusion, two reviewers independently performed data extraction following a predefined and standardized protocol to ensure consistency and minimize potential errors. Extracted data included essential study details such as the first author, year of publication, study design, characteristics of the participant population, descriptions of experimental and control groups, duration of follow-up (where applicable), and primary and secondary outcomes related to microbiological and periodontal measures. Special attention was given to detailed technical parameters of the indocyanine green-mediated photodynamic therapy, including the ICG concentration, light source specifications (wavelength, power output, energy density), method of application, and any adjunctive treatments combined with photodynamic therapy. Procedural aspects, such as treatment duration, irradiation technique, and frequency of application, were also systematically recorded to support comprehensive cross-study comparisons.

### 2.7. Study Selection

In accordance with the PRISMA 2020 guidelines, the process of study selection is outlined in Figure 1. The systematic search across multiple databases initially identified 161 records. After the removal of duplicates, 98 unique articles remained. Through screening of titles and abstracts, 16 studies were deemed potentially relevant and were selected for full-text review. All 16 studies met the inclusion criteria and were incorporated into the final analysis. These randomized controlled trials, all published within the last decade, specifically investigated the therapeutic efficacy of indocyanine green (ICG)-mediated photodynamic therapy in the management of periodontitis. Table 4 presents the geographical distribution of the included studies.

## 3. Results

### 3.1. Data Presentation

The structured presentation of study data in Table 4, Table 5, Table 6 and Table 7 is intended to highlight key patterns across clinical and methodological variables. Emphasis is placed not only on outcome measures but also on contextual factors, such as geography, laser parameters, and photosensitizer characteristics, which may explain inter-study variability. Given the heterogeneity in healthcare systems, patient populations, and practice settings, geographical context is essential to understanding local feasibility and treatment outcomes. For instance, differences in clinical guidelines, availability of laser technology, and training may contribute to observed discrepancies in efficacy across countries. Presenting this data transparently enables better interpretation of generalizability and potential barriers to clinical implementation.

### 3.2. Overview of Study Characteristics

The geographic distribution of the included studies is not incidental; it reflects global research interest and application diversity of ICG-mediated photodynamic therapy. Studies were conducted across Europe, Asia, South America, and the Middle East, indicating broad international uptake. However, the concentration of trials in certain regions, such as India and Saudi Arabia, may influence outcomes due to differences in baseline disease prevalence, healthcare access, or operator expertise. By identifying where these studies originate, readers gain insight into both the potential applicability of findings across diverse populations and the need for further validation in underrepresented regions, such as North America and Africa. This geographic lens also helps policymakers and clinicians assess transferability to their local contexts.

### 3.3. Main Study Outcomes

Indocyanine green-mediated antimicrobial photodynamic therapy (ICG-aPDT) has shown varying degrees of clinical and microbiological effectiveness as an adjunct in periodontal treatment. Al-Momani et al. (2021) [29] demonstrated that ICG-aPDT significantly improved probing depth (PD), clinical attachment level (CAL), bleeding on probing (BOP), and inflammatory markers across diabetic and non-diabetic groups, with sustained benefits in non-diabetics and well-controlled type 2 diabetes mellitus (T2DM). Annunziata et al. (2023) [30] found that while both test and control groups improved following full-mouth ultrasonic debridement (FMUD), ICG-aPDT led to greater reductions in deep pocket PD and key pathogens, such as *Aggregatibacter actinomycetemcomitans* and *Parvimonas micra*. Similarly, Cetiner et al. (2024) [31] reported that adjunctive ICG-aPDT enhanced wound healing, reduced deep pocket depths, and promoted osteogenic marker expression following regenerative surgery. In maintenance patients, Chowdhury et al. (2024) [32] observed no significant benefit of ICG-aPDT over scaling and root planing (SRP) alone, whereas Costa et al. (2023) [33] found notable reductions in BOP and key pathogens despite no improvement in PD or CAL. Dalvi et al. (2019) [34] highlighted better healing and gingival outcomes with adjunctive ICG-aPDT after open flap debridement. Hayashi et al. (2023) [35] confirmed short-term bacterial reductions using transgingival ICG-aPDT, though effects were not sustained. Hill et al. (2019) [36] and Joshi et al. (2020) [37] both found modest or transient benefits, with Hill noting short-term fluid flow reduction and Joshi showing enhanced PD and CAL improvements. Monzavi et al. (2016) [38] reported significant inflammation reduction without attachment gain, while Niazi et al. (2020) [39] observed superior CAL and PD outcomes versus Salvadora persica gel or SRP alone, though BOP reduction was greater with herbal therapy. Qamar et al. (2021) [40] demonstrated ICG-aPDT’s superiority over Aloe vera gel in improving inflammatory markers and clinical parameters. Sethi et al. (2020) [41] noted strong antimicrobial effects and significant CAL/PD improvement without gingival recession. Shingnapurkar et al. (2017) [42] found enhanced relative attachment level (RAL) and PPD reduction, and Srikanth et al. (2015) [43] confirmed significantly greater CAL and PD improvements with ICG-aPDT compared to diode laser or SRP alone, with no tissue damage. Finally, Sukumar et al. (2020) [44,45,46] demonstrated consistent clinical and microbiological superiority of ICG-aPDT over SRP, including effective suppression of multiple pathogens, such as *P. gingivalis* and *T. forsythia*. These findings suggest that while clinical efficacy varies, ICG-aPDT is generally safe and may offer enhanced benefits in deep or inflamed pockets, particularly when repeated or combined with regenerative or surgical approaches.

Although several studies reported statistically significant reductions in probing depth ranging from 0.5 to 1.2 mm, only reductions ≥1.0 mm are generally considered clinically meaningful, particularly in pockets ≥5 mm [47,48,49,50,51]. Smaller improvements, while statistically significant, may not translate into tangible clinical benefit in routine practice. ICG-aPDT resulted in statistically significant PD reductions (typically 0.5–1.2 mm); however, in many studies, these changes fell below the threshold for clinical relevance (≥1.0 mm), particularly in shallow or maintenance-phase pockets. While many studies included in this review demonstrated statistically significant improvements in PD and clinical attachment level (CAL), the magnitude of change often remained modest. According to established periodontal treatment guidelines, clinically meaningful PD reductions typically start at ≥1.0 mm, especially in deep pockets (≥5 mm) [47,48,49]. Reductions below this threshold, though statistically significant, may have a limited impact on long-term periodontal stability or tooth retention. Therefore, future studies should clearly distinguish between statistical and clinical significance and report responder rates or pocket closure as more meaningful clinical endpoints [50,51].

### 3.4. Characteristics of Light Sources Used in PDT

Light source parameters are central to the effectiveness of photodynamic therapy and must be analyzed in detail to understand differences in clinical efficacy. While ICG has an optimal absorption peak around 800 nm, diode lasers used in the included studies varied in wavelength (from 808 to 970 nm), power output, irradiation mode (continuous vs. pulsed), and application time. These variations directly influence the generation of reactive oxygen species (ROS) and, consequently, bacterial kill rates and host tissue responses. Moreover, discrepancies in fluence and exposure duration may explain inconsistencies in short- and long-term outcomes. By explicitly summarizing these laser characteristics in Table 6, we provide a mechanistic rationale for treatment variability and highlight the pressing need for protocol standardization in future trials. Understanding these physical parameters is also crucial for clinicians seeking to replicate PDT protocols in real-world practice.

## 4. Discussion

### 4.1. Results in the Context of Other Evidence

ICG-aPDT has demonstrated significant clinical benefits as an adjunct to conventional periodontal treatments, notably improving probing depth (PD) reduction and clinical attachment level (CAL) gain in both surgical and non-surgical contexts [29,30,31,34,37]. Studies consistently report superior outcomes in deep periodontal pockets (≥6 mm) when ICG-aPDT is added to scaling and root planing (SRP), particularly in terms of PD reduction and a reduction in bleeding on probing (BOP) [30,31,33,36]. ICG-aPDT enhances antimicrobial efficacy, leading to substantial reductions in key periodontal pathogens such as *P. gingivalis*, *A. actinomycetemcomitans*, and *T. forsythia* in various clinical scenarios [29,30,33,39,43]. Its benefits extend to diabetic patients, where ICG-aPDT showed consistent efficacy in reducing inflammation and improving periodontal parameters, even in poorly controlled glycemic states [29]. The adjunctive use of ICG-aPDT post-regenerative surgery was associated with accelerated wound healing and increased expression of osteogenic markers, suggesting potential benefits for tissue regeneration [31]. Multiple applications of ICG-aPDT provided more pronounced clinical and microbiological improvements compared to single-use protocols, underlining the importance of treatment frequency [43]. Despite some studies finding no significant intergroup differences, ICG-aPDT was valued for its non-invasiveness, safety profile, and lack of reported adverse effects across all trials [32,35,36]. The antimicrobial effect of ICG-aPDT appears short-lived in some cases, emphasizing the need for standardized protocols and longer-term evaluations to optimize clinical outcomes [35,38]. Furthermore, ICG’s ability to perform effectively under hypoxic conditions within periodontal pockets positions it as a favorable photosensitizer compared to alternatives [29,33,39]. Overall, while heterogeneity in protocols limits broad generalizations, the evidence supports ICG-aPDT as a promising, safe, and effective adjunct in modern periodontal therapy [29,30,31,32,33,34,35,36,37,38,39,40,41,42,43].

The efficacy of ICG-mediated PDT is highly dependent on the characteristics of the light source used, particularly wavelength, power output, energy density, and exposure time. ICG exhibits peak absorption near 800 nm, making near-infrared (NIR) lasers or diode lasers ideal for optimal activation and deeper tissue penetration [25,26,27,28,29,30,31,32]. Studies suggest that light sources with lower power settings and continuous wave modes may reduce thermal effects while maintaining antimicrobial efficacy, whereas pulsed modes might enhance ROS generation without damaging host tissues [33,34,35]. Further comparative studies are warranted to determine the ideal light delivery parameters that maximize therapeutic outcomes while minimizing adverse effects. While most clinical trials to date have not stratified outcomes based on ethnicity, future investigations should consider potential differences in treatment response among diverse ethnic populations, given known genetic and immunologic variations that may influence periodontal disease progression and healing capacity [35,36,37,38,39,40,41,42,43]. Moreover, ICG-mediated PDT has shown broad-spectrum antimicrobial effects against several key periodontal pathogens, including *P. gingivalis*, *A. actinomycetemcomitans*, *T. forsythia*, and *T. denticola*. However, pathogen-specific susceptibility and resistance patterns should be further explored, as preliminary evidence suggests differential sensitivity to PDT across microbial species. Understanding these variations could support more personalized and effective antimicrobial strategies in periodontal care [35,36,37,38,39,40,41,42,43].

Photodynamic therapy (PDT) has emerged as a promising adjunctive modality for periodontal treatment, with indocyanine green (ICG) gaining significant attention as a photosensitizer in recent years [44]. Indocyanine green possesses several pharmacological properties that make it particularly suitable for periodontal photodynamic therapy [45,46]. As a water-soluble tricarbocyanine dye approved by the U.S. Food and Drug Administration for clinical use in medical imaging, ICG exhibits favorable characteristics including high photostability, low toxicity, and strong absorption of near-infrared light around 800 nm wavelength [47,48]. These properties contribute to its growing popularity in periodontal applications, where targeted antimicrobial effects are crucial for effective management of pathogenic biofilms [46,47,48]. The fundamental mechanism of ICG-mediated PDT involves the application of the photosensitizer to periodontal pockets, followed by activation with a specific wavelength of light. Upon activation, ICG undergoes a photochemical reaction that generates reactive oxygen species, which exert potent cytotoxic effects on bacterial cell walls and membranes, ultimately leading to bacterial cell death. This process offers a targeted approach to eliminating periodontal pathogens while minimizing collateral damage to surrounding tissues [48,49]. Multiple clinical studies have investigated the efficacy of ICG-mediated PDT as an adjunct to scaling and root planing (SRP) in periodontitis management.

One of the most significant advantages of ICG as a photosensitizer is its absorption spectrum in the near-infrared range around 800 nm [35,36,37,38,39,40,41,42,43]. This property allows for deeper tissue penetration compared to photosensitizers that work with visible light wavelengths, enabling more effective targeting of subgingival pathogens located within periodontal pockets and tissues [35,36,37,38,39,40,41,42,43]. Furthermore, ICG demonstrates an excellent safety profile, with minimal risk of systemic effects or adverse reactions. Unlike antibiotics, which can contribute to antimicrobial resistance and potentially disrupt the commensal microbiota, ICG-mediated PDT offers targeted antimicrobial action with a low risk of developing resistance [39]. This aspect is particularly important in contemporary periodontal care, where antibiotic stewardship is increasingly emphasized [40]. Multiple studies have demonstrated ICG’s efficacy against periodontal pathogens, including those associated with peri-implantitis [38,39,40,41,42,43,44,45,46,47,48,49,50,51,52]. Beyond its antimicrobial effects, ICG-mediated PDT may offer additional anti-inflammatory benefits. Studies have shown reductions in inflammatory biomarkers following treatment, suggesting a multifaceted therapeutic approach that addresses both the microbial and inflammatory components of periodontal disease [53,54,55].

This dual action represents a significant advantage over conventional mechanical debridement, which primarily targets the bacterial biofilm without directly modulating the host inflammatory response [53]. Despite promising results in many studies, the clinical effectiveness of ICG-mediated PDT shows some inconsistency across different investigations. The additional benefit of ICG-PDT over SRP alone was limited to a transient reduction in sulcus fluid flow rate [37]. This suggests that the adjunctive value of ICG-PDT might not always translate to substantial long-term clinical advantages compared to conventional treatment approaches.

The lack of standardized protocols represents another significant limitation in ICG-mediated PDT. Variations in ICG concentration, application method, light parameters (wavelength, power output, energy density), exposure time, and treatment area can all influence treatment outcomes. This lack of standardization makes it difficult to compare results across studies and establish definitive guidelines for clinical practice.

These requirements may present barriers to widespread adoption, particularly in resource-limited settings. Additionally, the procedure typically extends treatment time, which may impact practice workflow and patient acceptance. When comparing ICG to other photosensitizers, such as methylene blue (MB), several differences emerge. A microbiological study on phototherapy of gingivitis using MB demonstrated total suppression of pathogenic flora after a 3 min exposure to the dye solution followed by a 20 s treatment with a red light-emitting toothbrush. While this study used MB rather than ICG, it provides a useful point of comparison regarding photosensitizer efficacy. MB operates at shorter wavelengths (around 663 nm) compared to ICG (around 800 nm), resulting in different tissue penetration profiles [56,57,58,59].

The red light used with MB has less tissue penetration than the near-infrared light used with ICG, potentially limiting its effectiveness in deeper periodontal pockets. However, MB has demonstrated significant antimicrobial effects in gingivitis treatment, suggesting that both photosensitizers have valid applications depending on the specific clinical scenario. Current evidence suggests that ICG-mediated PDT offers promising adjunctive benefits in periodontal therapy, but several knowledge gaps remain. Future research should focus on establishing standardized protocols, optimizing dosage and application methods, and investigating the long-term effects of repeated applications. Additionally, comparative studies directly evaluating different photosensitizers within the same experimental design would provide valuable insights into their relative efficacy and appropriate clinical applications [60,61,62,63,64,65].

Indocyanine green as a photosensitizer in periodontal photodynamic therapy presents several advantages, including excellent tissue penetration, a favorable safety profile, effective antimicrobial action, and potential anti-inflammatory effects [35,36,37,38,39,40,41,42,43]. However, limitations such as inconsistent clinical outcomes, lack of standardized protocols, temporary effects on inflammatory biomarkers, and practical implementation challenges must be considered [35,36,37,38,39,40,41,42,43]. The current evidence suggests that ICG-mediated PDT may serve as a valuable adjunct to conventional periodontal therapy, particularly in cases where mechanical debridement alone might be insufficient. However, clinicians should approach this modality with a clear understanding of both its potential benefits and limitations. Further research with standardized protocols and long-term follow-up periods is needed to establish definitive guidelines for the optimal use of ICG in periodontal applications.

The wide variation in ICG concentration (ranging from 0.1 to 5 mg/mL) and laser power output (100–2000 mW) across studies likely contributed to the heterogeneity of outcomes observed, as these parameters critically affect the photothermal and photodynamic efficacy of treatment; however, due to insufficient stratified reporting, a direct dose–response relationship could not be formally analyzed in this review. Future trials should employ standardized or at least well-justified ICG and laser dosimetry parameters and explicitly evaluate their impact on clinical and microbiological endpoints to optimize treatment protocols.

The greater antimicrobial efficacy of ICG-aPDT against *P. gingivalis* and *A. actinomycetemcomitans*, observed across multiple studies, may be attributed to both the photochemical properties of ICG and the structural vulnerabilities of these Gram-negative pathogens [17,30,37,53]. ICG absorbs near-infrared light (~800 nm), which penetrates deeply into tissue and, upon activation, generates both reactive oxygen species (ROS) and localized heat (photothermal effect) [7,53]. These mechanisms disrupt bacterial membranes, denature proteins, and damage nucleic acids. *P. gingivalis* and *A. actinomycetemcomitans* are particularly susceptible due to their thin peptidoglycan layer and high membrane lipid content, which enhance light absorption and thermal sensitivity [30,37]. Additionally, ICG’s anionic and amphiphilic nature promotes binding to bacterial membranes and biofilm matrices, facilitating targeted phototoxic effects [53]. The short incubation time and low photobleaching tendency of ICG further support efficient ROS-mediated killing within subgingival pockets [7,53]. In contrast, Gram-positive species or facultative aerobes with thicker cell walls may be less susceptible under equivalent conditions [37].

### 4.2. Limitations of the Evidence

The current body of evidence supporting the use of ICG-aPDT as an adjunctive treatment for periodontitis is limited by several important factors. Despite the inclusion of only randomized controlled trials, substantial heterogeneity exists across studies in terms of ICG concentrations, laser wavelengths, power outputs, irradiation times, and treatment frequencies. This variability makes it difficult to directly compare outcomes or establish universally applicable treatment protocols. Additionally, most studies included relatively short follow-up periods, typically 1 to 6 months, limiting our understanding of the long-term effectiveness and stability of ICG-aPDT outcomes. While several trials demonstrated promising improvements in probing depth and microbial reduction, inconsistencies in reported outcomes and a lack of standardized microbiological assessment methods further weaken the generalizability of the results. Moreover, many studies lacked detailed reporting on potential confounding factors, such as smoking status, oral hygiene compliance, or systemic conditions beyond diabetes, which could have influenced treatment efficacy. Few studies directly compared ICG with other photosensitizers, making it difficult to definitively determine its relative superiority. Lastly, while safety was consistently reported, adverse effects may have been underreported due to limited sample sizes or short observation windows. Collectively, these limitations underscore the need for larger, long-term, multicenter trials with standardized protocols to more definitively assess the clinical value of ICG-aPDT in periodontal therapy.

### 4.3. Limitations of the Review Process

While this systematic review provides a synthesis of the available evidence on ICG-aPDT for the treatment of periodontitis, several limitations of the review process must be acknowledged. Firstly, although only randomized controlled trials (RCTs) were included to ensure methodological rigor, the clinical heterogeneity among these studies, such as differences in treatment protocols, ICG concentrations, laser parameters, application techniques, and follow-up durations, hindered the ability to perform a meta-analysis and limited the comparability of the findings. Secondly, most studies lacked long-term follow-up data, thereby restricting insights into the durability of treatment outcomes. Thirdly, language bias may have influenced the findings, as only studies published in English were considered, potentially omitting relevant trials published in other languages. Furthermore, despite the low risk of bias identified in quality assessments, reporting inconsistencies in microbiological data and selective outcome measures in some trials could have introduced a degree of reporting bias. Finally, the exclusion of the gray literature and unpublished data may have contributed to publication bias. These factors collectively highlight the need for future research to adhere to standardized methodologies and reporting frameworks to enhance comparability and strengthen clinical recommendations for ICG-aPDT in periodontal therapy.

### 4.4. Implications for Practice, Policy, and Future Research

The findings of this systematic review support the integration of ICG-aPDT as a safe and effective adjunct to conventional periodontal treatments, particularly in managing deep periodontal pockets and in systemically compromised patients, such as those with diabetes mellitus. In clinical practice, ICG-aPDT offers a minimally invasive, well tolerated option with demonstrated antimicrobial and anti-inflammatory effects, making it a valuable addition to periodontal maintenance and regenerative protocols. However, due to variability in treatment protocols and limited long-term data, clinicians should apply ICG-aPDT with caution and ensure patients are informed about its adjunctive role rather than a replacement for mechanical debridement. From a policy perspective, current evidence does not yet support the widespread recommendation of ICG-aPDT in clinical guidelines, largely due to the absence of standardized dosing, irradiation parameters, and frequency of application. To move toward broader clinical adoption, regulatory and professional bodies should prioritize the development of consensus-based guidelines grounded in high-quality, reproducible evidence. Future research should focus on multicenter, long-duration randomized controlled trials with clearly defined protocols and standardized outcome measures, including microbiological, inflammatory, and clinical parameters. Comparative studies evaluating ICG against other photosensitizers under uniform conditions would provide critical insights into its relative efficacy. Investigations into cost effectiveness, patient-reported outcomes, and implementation feasibility in diverse clinical settings are also essential to support evidence-based decision-making. Ultimately, addressing these research gaps will facilitate the optimization and broader clinical integration of ICG-aPDT in modern periodontal therapy.

## 5. Conclusions

This systematic review confirms that indocyanine green-mediated photodynamic therapy (ICG-aPDT) is a safe and effective adjunct to conventional periodontal treatments, particularly in deep pockets and systemically compromised patients. While most studies demonstrated statistically significant clinical and microbiological improvements with adjunctive ICG-aPDT, inconsistent outcomes in some trials may reflect variability in treatment protocols, photosensitizer formulation, baseline disease severity, and timing of outcome assessment, underscoring the need for standardized methodologies and stratified analyses in future research. ICG-aPDT holds strong potential as a non-invasive tool in modern periodontal therapy, especially when tailored to well-defined clinical protocols.

## Figures and Tables

**Table 1:** Search syntax used in the study.

Source	Search Term	Filters	Number of Results
PubMed	(“Photodynamic Therapy” [Mesh] OR “Photodynamic Therapy” [tiab]) AND (“Indocyanine Green” [Mesh] OR “Indocyanine Green” [tiab]) AND (“Periodontitis” [Mesh] OR “Periodontitis” [tiab])	Randomized Controlled Trial Years: 2015–2025	17
Embase	(“photodynamic therapy”/exp OR “photodynamic therapy”: ti,ab) AND (“indocyanine green”/exp OR “indocyanine green”: ti,ab) AND (“periodontitis”/exp OR “periodontitis”: ti,ab)	Article Years: 2015–2025	45
Scopus	(TITLE-ABS-KEY (“Photodynamic Therapy”)) AND (TITLE-ABS-KEY (“Indocyanine Green”)) AND (TITLE-ABS-KEY (“Periodontitis”)) AND (LIMIT-TO (DOCTYPE, “ar”))	Article Years: 2015–2025	56
Cochrane	(“Photodynamic Therapy”:ti,ab OR “Photodynamic Treatment”:ti,ab) AND (“Indocyanine Green”:ti,ab) AND (“Periodontitis”: ti,ab OR “Periodontal Disease”: ti,ab)	Years: 2015–2025	43

**Table 2:** Selection criteria used in the study.

Inclusion Criteria	Exclusion Criteria
Only randomized controlled trials (RCTs) investigating the antimicrobial and/or clinical effects of indocyanine green (ICG)-mediated photodynamic therapy in patients with periodontitis. Studies evaluating bacterial reduction or eradication outcomes following ICG-based treatment. RCTs where ICG serves as the primary photosensitizer within the photodynamic therapy protocol. Research explicitly reporting microbial outcomes, such as bacterial counts, specific bacterial species, or shifts in microbial composition. Studies comparing the effectiveness of ICG-assisted therapy to conventional periodontal therapies or other adjunctive modalities. Trials incorporating follow-up assessments to evaluate the durability of antimicrobial effects and periodontal health outcomes over time.	Non-peer-reviewed publications, including conference abstracts, case reports, editorials, opinion articles, book chapters, and unpublished theses. Articles published in languages other than English. Duplicate publications or multiple reports based on the same patient cohort without presenting new or distinct findings. Research unrelated to periodontitis or not focused on antimicrobial applications of photodynamic therapy. Studies lacking a control or comparison group necessary to contextualize treatment outcomes. Investigations where ICG was not the primary photosensitizer used in the intervention. Studies evaluating alternative photosensitizers or adjunctive therapies without specifically assessing ICG efficacy. In vitro studies employing overly simplified or artificial models with limited relevance to clinical periodontal treatment. Research targeting pathogens or conditions not relevant to periodontal disease.

**Table 3:** The results of the quality assessment and risk of bias across the studies.

	1	2	3	4	5	6	7	8	9	10	11	12	13	14	Total Score	Risk of Bias
Al-Momani et al., 2021 [29]	1	1	1	1	1	1	1	1	1	1	1	1	1	1	14	Low
Annunziata et al., 2023 [30]	1	1	1	1	1	1	1	1	1	1	1	1	1	1	14	Low
Cetiner et al., 2024 [31]	1	1	1	1	1	1	1	1	1	1	1	1	1	1	14	Low
Chowdhury et al., 2024 [32]	1	1	1	1	1	1	1	1	1	1	1	1	1	1	14	Low
Costa et al., 2023 [33]	1	1	1	1	1	1	1	1	1	1	1	1	1	1	14	Low
Dalvi et al., 2019 [34]	1	1	1	1	1	1	1	1	0	1	1	1	1	1	13	Low
Hayashi et al., 2023 [35]	1	1	1	1	1	1	1	1	1	1	1	1	1	1	14	Low
Hill et al., 2019 [36]	1	1	1	1	1	1	1	1	1	1	1	1	1	1	14	Low
Joshi et al., 2020 [37]	1	1	1	1	1	1	1	1	1	1	1	1	1	1	14	Low
Monzavi et al., 2016 [38]	1	1	1	1	1	1	1	1	0	1	1	1	1	1	13	Low
Niazi et al., 2020 [39]	1	1	1	1	1	1	1	1	1	1	1	1	1	1	14	Low
Qamar et al., 2021 [40]	1	1	1	1	1	1	1	1	1	1	1	1	1	1	14	Low
Sethi et al., 2020 [41]	1	1	1	1	1	1	1	1	1	1	1	1	1	1	14	Low
Shingnapurkar et al., 2017 [42]	1	1	1	1	1	1	1	1	1	1	1	1	1	1	14	Low
Srikanth et al., 2015 [43]	1	1	1	1	1	1	1	1	1	1	1	1	1	1	14	Low
Sukumar et al., 2020 [44]	1	1	1	1	1	1	1	1	1	1	1	1	1	1	14	Low

**Table 4:** A geographic distribution of the included research.

Study	Country
Al-Momani et al., 2021 [29]	Saudi Arabia
Annunziata et al., 2023 [30]	Italy/Spain
Cetiner et al., 2024 [31]	Turkey/Switzerland
Chowdhury et al., 2024 [32]	India
Costa et al., 2023 [33]	Brazil
Dalvi et al., 2019 [34]	India/UK/Italy
Hayashi et al., 2023 [35]	Japan
Hill et al., 2019 [36]	Germany
Joshi et al., 2020 [37]	India
Monzavi et al., 2016 [38]	Iran/Germany
Niazi et al., 2020 [39]	Saudi Arabia/Pakistan
Qamar et al., 2021 [40]	Saudi Arabia/Pakistan/Malaysia
Sethi et al., 2020 [41]	India
Shingnapurkar et al., 2017 [42]	India
Srikanth et al., 2015 [43]	India
Sukumar et al., 2020 [44]	India

**Table 5:** Summary of the principal results and study details.

Author and Year	Study Groups	Outcomes
Al-Momani et al., 2021 [29]	3 glycemic groups: non-diabetic (*n* = 17), well-controlled T2DM (*n* = 17), and poorly controlled T2DM (*n* = 16). Using a split-mouth design, each patient received RSD alone (control) on one site and ICG-aPDT with RSD (test) on the other, allowing intra-patient comparison within each group.	- ICG-aPDT as an adjunct to RSD significantly improved clinical, microbiological, and immune-inflammatory outcomes in stage III, grade C periodontitis. - Effective in both well-controlled and poorly controlled T2DM patients, as well as non-diabetics. - Led to greater reductions in BOP, PD, and CAL compared to RSD alone. - Improvements sustained at 3 and 6 months in well-controlled T2DM and non-diabetics. - Improvements observed at 3 months in poorly controlled T2DM. - Caused a greater reduction in *P. gingivalis* and *T. forsythia* levels. - Reduced inflammatory cytokines IL-17 and IFN-γ in gingival crevicular fluid more effectively than RSD alone. - Superior efficacy regardless of glycemic control, suggesting effectiveness even in hypoxic environments, a potential advantage in diabetic patients.
Annunziata et al., 2023 [30]	24 patients with generalized stage III, grade B periodontitis were randomly assigned to test and control groups (*n* = 12 each). All received FMUD. One week later, the test group underwent ICG-aPDT with an 810 nm diode laser at sites with PD > 4 mm, repeated at four weeks. The control group received identical treatment with the laser in off mode, enabling evaluation of active ICG-aPDT versus placebo alongside FMUD.	- ICG-aPDT was evaluated as an adjunct to FMUD in periodontitis treatment. - Although both test and control groups showed significant clinical improvements at 3 and 6 months, the ICG-aPDT group demonstrated a significantly greater reduction in PD, specifically in initially deep pockets (≥6 mm) and a higher percentage of closed pockets (PD ≤ 4 mm and no bleeding on probing) at 6 months. - Microbiological analysis revealed a significantly greater reduction in *Aggregatibacter actinomycetemcomitans* and *Parvimonas micra* in the ICG-aPDT group at 3 months, though this effect was not sustained at 6 months. - Overall, the adjunctive use of ICG-aPDT provided limited clinical and microbiological benefits beyond FMUD alone, with improvements largely restricted to deeper periodontal pockets.
Cetiner et al., 2024 [31]	48 systemically healthy patients with stage III/IV grade C periodontitis were randomly assigned to four groups after regenerative surgery: control (saline irrigation), aPDT (ICG-aPDT with a 970 ± 15 nm diode laser), photobiomodulation (626 nm LED), and ozone therapy.	- ICG-aPDT, used adjunctively with regenerative surgery in stage III/IV grade C periodontitis, showed significantly better outcomes in deep pockets (PD > 7 mm), with greater CAL gains and PD reductions at 6 months compared to controls. - It also promoted better early wound healing, with no membrane exposure and consistently high EHI scores. - Molecular analysis revealed increased expression of osteogenic markers (osterix, Nell-1), indicating a potential role in bone regeneration.
Chowdhury et al., 2024 [32]	25 patients in periodontal maintenance (24 completed) received full-mouth SRP. One side (test) also underwent ICG-aPDT with an 810 nm diode laser after SRP and again at 14 days; the other side (control) received SRP and a sham laser. Site allocation was randomized, with blinding of participants, investigators, and assessors.	- ICG-aPDT was evaluated as an adjunct to SRP in periodontal maintenance patients with residual pockets. Both groups showed significant improvements in Ps, CAL, plaque index, and bleeding scores at 3 months, but no significant differences were found between them. - Similarly, reductions in key pathogens (*A. actinomycetemcomitans*, *P. gingivalis*, *T. forsythia*, *F. nucleatum*) were observed in both groups without intergroup significance. - While ICG-aPDT offered no added clinical or microbiological benefit over SRP alone, its ease of use and potential warrant further study with standardized protocols and larger samples.
Costa et al., 2023 [33]	24 periodontal maintenance patients with residual pockets (PD ≥5 mm) in contralateral quadrants were enrolled. Sites were randomized to test (SRP + ICG-aPDT using a 909 nm diode laser) or control (SRP + sham aPDT). Treatment was repeated after 15 days, enabling within-subject comparison of clinical and microbiological effects.	- ICG-aPDT was assessed as an adjunct to SRP in residual pockets (PD ≥5 mm) during periodontal maintenance. - While both groups showed clinical improvement over six months, ICG-aPDT led to significantly greater reductions in BOP, PI-SA, and levels of *P. gingivalis* and *A. actinomycetemcomitans*. No significant differences were observed in plaque index, PD, or CAL. - The treatment was safe, and its benefits were attributed to ICG’s photothermal action, effective even in the hypoxic conditions of periodontal pockets.
Dalvi et al., 2019 [34]	20 systemically healthy chronic periodontitis patients provided two sites each (40 total), randomized to test (OFD + single-session ICG-aPDT with 1 mg/mL ICG and 810 nm diode laser) or control (OFD alone). All underwent full-mouth SRP before surgery. Clinical outcomes were assessed at baseline and three months to evaluate ICG-aPDT’s adjunctive benefits.	- A single session of ICG-aPDT with an 810 nm diode laser was used adjunctively with OFD in treating chronic periodontitis. - The ICG-aPDT group showed significantly greater improvements in RAL, RGML, and GI, indicating better healing and gingival health. - While both groups improved in PPD, PI, and GBI, gains were more pronounced with ICG-aPDT. - No adverse effects occurred, and the treatment reduced gingival recession and enhanced tissue healing.
Hayashi et al., 2023 [35]	40 periodontal maintenance patients with ≥5 mm pockets were randomized into two groups (*n* = 20 each). The test group received transgingival aPDT with an 810 nm diode laser and ICG-nano/c photosensitizer; the control group received laser irradiation with PBS placebo. This setup evaluated the short-term bactericidal effects and safety of transgingival ICG-aPDT.	- Transgingival ICG-aPDT was evaluated in supportive periodontal therapy patients with residual pockets. - It reduced viable bacterial colony counts immediately after treatment, with more patients achieving reductions to ≤50% and ≤10% compared to controls. - However, this effect was not sustained after one week, and no significant changes were seen in bacterial DNA load or composition. - The treatment was safe, painless, and caused no soft tissue damage. - The study concluded that while transgingival ICG-aPDT offers short-term bactericidal effects, its clinical efficacy was less than expected based on in vitro results.
Hill et al., 2019 [36]	20 medically healthy, non-smoking chronic periodontitis patients received full-mouth SRP. In each, two quadrants were randomly assigned to the test group (SRP + ICG-aPDT using an 808 nm diode laser with 0.1 mg/mL ICG) and two to the control group (SRP alone). This split-mouth design enabled direct comparison of adjunctive ICG-aPDT effects.	- ICG-aPDT was assessed as an adjunct to SRP using a split-mouth design. - Both groups showed significant improvements in BOP, PD, and RAL at 3 and 6 months, with no significant differences except for a transient reduction in SFFR in the ICG-aPDT group at two weeks. - While both groups reduced *P. gingivalis*, ICG-aPDT led to greater, though modest, reductions in *P. intermedia* and *T. denticola*. - A single ICG-aPDT application did not provide sustained clinical or microbiological benefits over SRP alone but was safe and well tolerated.
Joshi et al., 2020 [37]	29 patients with chronic generalized periodontitis had two contralateral quadrants randomized to control (SRP alone) or test (SRP + ICG-aPDT with 1 mg/mL ICG and an 810 nm diode laser). Clinical parameters were assessed at baseline and 3 months to evaluate the added efficacy of ICG-aPDT in non-surgical treatment.	- ICG-aPDT, used as an adjunct to SRP in chronic periodontitis, led to significantly greater reductions in PPD and greater CAL gains over 3 months compared to SRP alone. - Both groups showed similar improvements in PI and mSBI, with no significant differences. No adverse effects were reported. - The study concluded that ICG-aPDT enhances SRP efficacy, especially for PPD and CAL, though further research with microbiological analysis and extended follow-up is needed.
Monzavi et al., 2016 [38]	50 chronic periodontitis patients were randomized into test (*n* = 25; SRP + ICG-aPDT with 810 nm diode laser) and control (*n* = 25; SRP + sham saline and inactive laser) groups. aPDT was repeated on days 7, 17, and 27. Clinical outcomes were assessed at baseline, 1 month, and 3 months to evaluate ICG-aPDT’s added benefits in non-surgical therapy.	- ICG-aPDT, used with SRP in chronic periodontitis, led to significantly greater reductions in BOP, PPD, and FMBS at 1 and 3 months compared to SRP alone. - However, no significant differences were found for CAL, PI, or FMPS. - While it effectively resolved inflammation and reduced deep pockets, it did not enhance attachment gain or plaque control. - The treatment was safe and well tolerated, with no adverse effects reported.
Niazi et al., 2020 [39]	73 chronic periodontitis patients were randomized into three groups: Group I (SRP + ICG-aPDT with 810 nm diode laser), Group II (SRP + *Salvadora persica* gel), and Group III (SRP alone). This design enabled comparison of clinical and inflammatory outcomes of PDT and herbal gel therapy versus standard treatment.	- ICG-aPDT, as an adjunct to SRP, reduced PD in moderate and deep periodontal pockets and improved CAL at 3 and 6 months compared to SRP alone and *Salvadora persica* gel. - While all groups showed improvements in plaque index and BOP, BOP reduction was greater with SP gel. ICG-aPDT notably lowered IL-6 levels at 3 months, though TNF-α reduction was more marked with SP gel.
Qamar et al., 2021 [40]	150 chronic periodontitis patients were randomized into three groups (*n* = 50 each): Group 1 received SRP alone, Group 2 received SRP + ICG-aPDT (810 nm diode laser), and Group 3 received SRP + AV gel applied to pockets for one hour. Clinical and inflammatory markers were measured at baseline, 3, and 6 months to compare adjunctive treatment efficacy.	- ICG-aPDT, used alongside SRP in chronic periodontitis, significantly improved PI, reduced PD, and increased CAL at both 3 and 6 months compared to SRP alone. - It also lowered IL-6, IL-8, and TNF-α levels in gingival crevicular fluid, with effects sustained at 6 months. - While both ICG-aPDT and Aloe vera gel were effective, ICG-aPDT showed greater benefits in CAL gain and PD reduction, highlighting its potential as a minimally invasive, effective adjunctive therapy.
Sethi et al., 2020 [41]	30 systemically healthy chronic periodontitis patients were randomized into control (SRP alone) and test (SRP + ICG-aPDT with 5 mg/mL ICG and 810 nm diode laser) groups (*n* = 15 each). Clinical and microbiological outcomes were assessed at baseline and 3 months to compare the effects of adjunctive ICG-aPDT versus SRP alone.	- ICG-aPDT adjunctive to SRP significantly improved SBI, PPD, and CAL after three months compared to SRP alone, though both groups showed similar PI improvements and no difference in GR. The ICG-aPDT group also had marked reductions in bacterial colonies and Gram-negative rods, indicating strong antimicrobial effects. - No adverse effects were reported.
Shingnapurkar et al., 2017 [42]	33 patients with untreated chronic periodontitis were included, and each patient contributed two contralateral sites with PPD > 5 mm. These sites were randomly assigned into two groups: Group A (control group) received SRP alone, while Group B (test group) received SRP followed by adjunctive ICG-aPDT using an 810 nm diode laser. Both groups were assessed at baseline, 1 month, and 3 months for clinical parameters including PI, GI, PPD, and RAL. This design allowed for intra-individual comparison of the additional benefits of ICG-aPDT.	- ICG-aPDT combined with SRP significantly improved clinical outcomes in chronic periodontitis compared to SRP alone. - At 3 months, the ICG-aPDT group showed greater PPD reduction and a significantly higher RAL gain (*p* = 0.007). - While both groups improved in PI and GI, intergroup differences were not significant. - ICG-aPDT was well tolerated, with no adverse effects, and selectively stained plaque without affecting teeth or restorations.
Srikanth et al., 2015 [43]	30 systemically healthy patients with chronic periodontitis were enrolled, with 27 completing the study. Each contributed one site per quadrant to three groups: SRP alone, SRP plus diode laser (810 nm, 0.7 W, 5 s), and SRP plus ICG-aPDT (5 mg/mL ICG with the same laser). This intra-individual design enabled comparison of bacterial viability, LDH levels, and clinical outcomes to evaluate the efficacy and safety of ICG-aPDT as an adjunct to non-surgical therapy.	- ICG-aPDT, adjunctive to SRP, reduced viable bacteria immediately post-treatment and maintained lower bacterial levels after one week compared to SRP or diode laser alone. - All groups improved in PD, CAL, PI, and MGI, but the ICG-aPDT group showed the greatest, statistically significant gains in CAL and PD. - LDH levels remained stable, indicating no tissue damage.
Sukumar et al., 2020 [44]	33 systemically healthy patients with chronic periodontitis affecting mandibular posterior sextants were treated using a split-mouth design. Test sites received SRP plus multiple ICG-aPDT applications (1 mg/mL ICG activated with an 810 nm diode laser at baseline, and weeks 1, 2, and 4), while control sites received SRP alone. This intra-individual comparison allowed evaluation of clinical and microbiological outcomes over 6 months.	- ICG-aPDT, used alongside SRP in chronic periodontitis, resulted in significantly greater reductions in PPD, CAL, PI, GI, and GBI at 3 and 6 months compared to SRP alone. - It also achieved superior reductions in key periodontal pathogens, including *P. gingivalis*, *A. actinomycetemcomitans*, *T. forsythia*, *F. nucleatum*, and *T. denticola.*

T2DM—type 2 diabetes mellitus; RSD—Root Surface Debridement; ICG-aPDT—indocyanine green-mediated antimicrobial photodynamic therapy; BOP—bleeding on probing; PD—probing depth; CAL—clinical attachment level; *T. forsythia*—*Tannerella forsythia*; IL-17—Interleukin-17; IFN-γ—interferon gamma; FMUD—full-mouth ultrasonic debridement; OFD—open flap debridement; RAL—relative attachment level; RGML—Relative Gingival Margin Level; GI—Gingival Index; PI—plaque index; GBI—Gingival Bleeding Index; SFFR—sulcus fluid flow rate; mSBI—Modified Sulcular Bleeding Index; FMBS—Full-Mouth Bleeding Score; FMPS—Full-Mouth Plaque Score; IL-6—Interleukin-6; TNF-α—Tumor Necrosis Factor-alpha; IL-8—Interleukin-8; GR—gingival recession; LDH—Lactate Dehydrogenase; MGI—Modified Gingival Index; AV—Aloe vera; ICG-nano/c—Indocyanine Green-encapsulated Chitosan-coated Nanoparticles; PBS—Phosphate-Buffered Saline; EHI—Early Wound Healing Index.

**Table 6:** Characteristics of the light source used.

Author and Year	Light Source	Wavelength	Operating Mode	Power Output (mW)	Irradiation Time (s)
Al-Momani et al., 2021 [29]	810 nm diode laser (A.R.C. laser GmbH, Nurnberg, Germany)	810	continuous mode	200	30—papilla 10—buccal and lingual side
Annunziata et al., 2023 [30]	810 nm diode laser unit (Fox ARC, Sweden & Martina, Due Carrare, Italy)	810	pulsed mode	300	30
Cetiner et al., 2024 [31]	970 ± 15 nm (SiroLaser Xtend; Sirona Dental Systems GmbH, Bensheim, Germany)	970 ± 15 nm	continuous mode	2000	180
Chowdhury et al., 2024 [32]	diode laser (Picasso AMD) 810	810	continuous mode,	2000	60
Costa et al., 2023 [33]	diode laser MM Optics, S.o Carlos, Brazil	909	continuous mode	500	5
Dalvi et al., 2019 [34]	diode laser (Picasso Lite, AMD Laser)	810	continuous mode	100 mW	30
Hayashi et al., 2023 [35]	diode laser (LIGHTSURGE SQUARE, Osada, Tokyo, Japan)	810 Å 20 nm		2000	40
Hill et al., 2019 [36]	diode laser (elexxion claros pico^®^, elexxion AG, Radolfzell, Germany)	808	pulsed	100	60
Joshi et al., 2020 [37]	diode laser	810		200	30
Monzavi et al., 2016 [38]	diode laser (A.R.C. laser GmbH, Nurnberg, Germany)	810	continuous mode		30
Niazi et al., 2020 [39]	GaAlAs diode laser (Picasso, AMD Lasers)	810		100	60
Qamar et al., 2021 [40]	GaAIAs laser diode (AMD Lasers; Indianapolis, IN, USA)	810		100	60
Sethi et al., 2020 [41]	diode laser (Biolase, Lake Forest, CA, USA)	810	continuous mode	800	60
Shingnapurkar et al., 2017 [42]	diode lasers	810	pulsed	100	30
Srikanth et al., 2015 [43]	diode laser	810	continuous wave	700	5
Sukumar et al., 2020 [44]		810		800	30

**Table 7:** Properties of ICG as a photosensitizer.

Property	Details
Chemical Name	Indocyanine green
Molecular Formula	C_43_H_47_N_2_NaO_6_S_2_
Molecular Weight	~774.96 g/mol
Structure	Tricarbocyanine dye with sulfonate groups for water solubility
Solubility	Soluble in water and plasma
Absorption Peak (λmax)	~780–810 nm (near-infrared region)
Emission Peak	~820–840 nm
Light Penetration	High tissue penetration due to NIR absorption
Quantum Yield (Φ)	Low (~0.01–0.05 in aqueous solutions)
Photostability	Moderate; can degrade under prolonged light exposure
Reactive Oxygen Species (ROS) Generation	Mainly Type I (radicals); limited Type II (singlet oxygen) activity
Aggregation Tendency	Tends to aggregate in aqueous media, reducing photodynamic efficacy
Targeting	Passive accumulation in tumors via the enhanced permeability and retention effect
Biocompatibility	Generally biocompatible and FDA approved for diagnostic use
Clearance	Rapid hepatic clearance; excreted via bile
Clinical Use	FDA approved for angiography; now being explored in PDT and imaging

Source: [29,30,31,32,33,34,35,36,37,38,39,40,41,42,43].
